# P-1189. In Vitro Interaction Between Amphotericin B and Oteseconazole: Exploring the Potential for Combination Therapy for Talaromycosis

**DOI:** 10.1093/ofid/ofaf695.1382

**Published:** 2026-01-11

**Authors:** Heera Natesan Sambath, Matthew Burke, Thi Mai Thu Nguyen, Emily Evans, Thi Hoa Ngo, Thuy Le

**Affiliations:** Duke University, Durham, NC; Duke University School of Medicine, Durham, North Carolina; Duke University School of Medicine, Durham, North Carolina; Emory University, Atlanta, Georgia; Pham Ngoc Thach University of Medicine, Ho Chi Minh City, Ho Chi Minh, Vietnam; Duke University School of Medicine, Durham, North Carolina

## Abstract

**Background:**

The mortality due to talaromycosis is up to 30% despite standard amphotericin B (AmB) therapy. While combination antifungal therapy has proven benefits in other mycoses, evidence for talaromycosis is lacking. Oteseconazole (OTZ), a newly FDA-approved azole, has low MIC against *Talaromyces marneffei* (Tm), a half-life of 20 weeks, a good safety profile, and is not metabolized by human cytochrome P450, collectively making it an exciting choice for prolonged consolidation and maintenance therapy for talaromycosis while minimizing drug interactions seen with current azoles.

**Figure 1:**
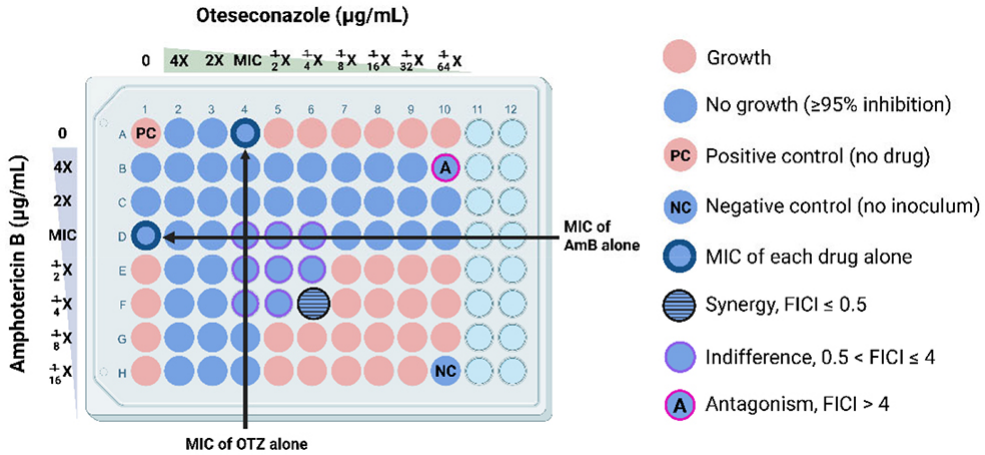
Drug-drug interaction between amphotericin B (AmB) and oteseconazole (OTZ) against Talaromyces marneffei assessed using the checkerboard assay. The 96-well plate contains 2-fold serial dilutions of AmB (2 to 0.03 µg/mL) and OTZ (0.25 to 0.0005 µg/mL). Positive control (pink, well A1) contains only Talaromyces marneffei and allows for uninhibited fungal growth. Negative control (blue, well H10) contains only the growth media RPMI-MOPs. Synergy is defined as a minimum of 4-fold reduction in the minimum inhibitory concentration (MIC) of the tested combination drugs compared to testing alone.

**Figure 2:**
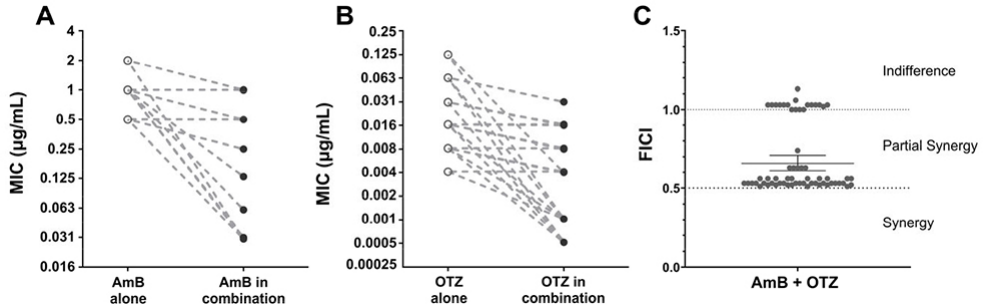
Checkerboard assay testing the interaction between amphotericin B (AmB) and oteseconazole (OTZ) combination against 62 Talaromyces marneffei clinical isolates. (A) Minimum inhibitory concentration (MIC) of AmB in combination with OTZ vs. AmB alone, (B) MIC of OTZ in combination with AmB vs. OTZ alone, and (C) Categorization of the AmB-OTZ interaction. The results showed a 4-fold reduction in MIC of AmB and OTZ when used in combination vs alone. Among the 62 tested isolates, 48 (77%) demonstrated partial synergy, while the remaining 14 (23%) isolates showed indifference.

**Methods:**

Using our newly developed colorimetric checkerboard assay, we investigated the *in vitro* interaction between AmB and OTZ against 62 Tm clinical isolates from Vietnam. A standard inoculum was tested on serial dilutions of AmB and OTZ in 96-well plates. We defined MICs as the lowest concentrations causing 95% growth inhibition. Drug interactions were classified by fractional inhibitory concentration index (FICI) as synergy (≤ 0.5), partial synergy (0.5–1), indifference (1–4), or antagonism ( > 4) (Figure 1). Checkerboard assay results were further validated in six isolates using time-kill assay to assess antifungal kinetics of AmB and OTZ alone and in combination.

**Figure 3:**
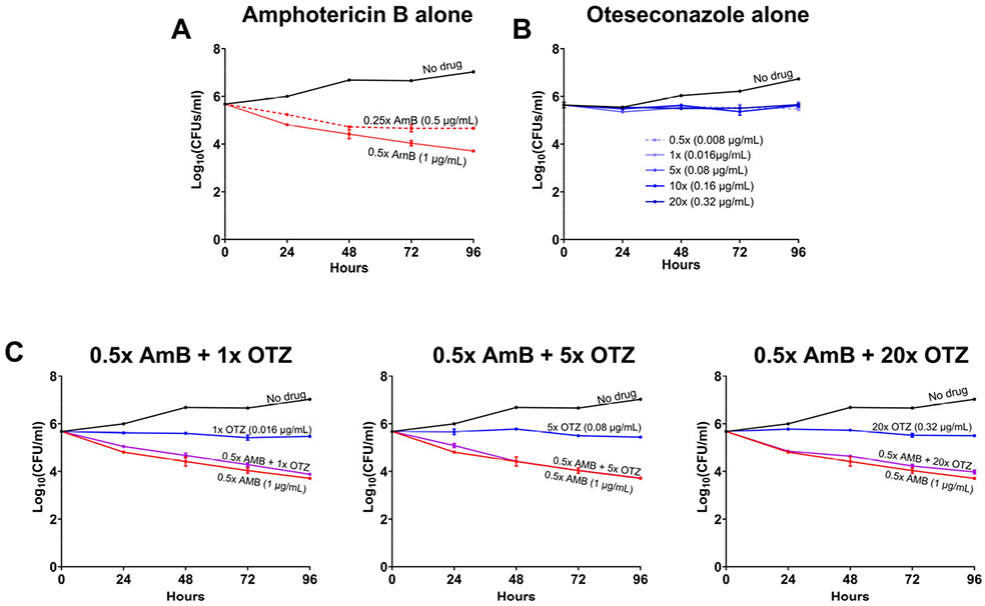
Time-kill assay to assess the antifungal kinetics of amphotericin B (AmB) and oteseconazole (OTZ) alone versus in combination against Talaromyces marneffei. (A) demonstrates concentration-dependent antifungal activity of AmB. (B) demonstrates concentration-independent antifungal activity of OTZ. The no-drug control curve contains only the fungal inoculum and allows for uninhibited fungal growth. (C) shows the antifungal effect of the combinations of sub-MIC concentration of AmB (0.5x MIC) with 1x, 5x, and 20x MIC of OTZ, demonstrating indifference interaction (i.e. < 2 log10 CFUs/mL reduction in the AmB-OTZ combination curve compared to AmB alone). Antagonism was not observed at all concentrations of OTZ.

**Results:**

Checkerboard Assay (Figure 2): The geometric mean (GM) MIC for AmB decreased from 1.01µg/mL to 0.12 µg/mL when combined with OTZ (≥ 4-fold decrease). GM MIC for OTZ decreased from 0.016 µg/mL to 0.004 µg/mL when combined with AmB (4-fold decrease). A majority of the isolates (48, 77%) showed partial synergy, while the remaining (14, 23%) showed indifference interaction.

Time-kill Assay (Figure 3): AmB exhibited concentration-dependent killing against Tm, while OTZ had a concentration-independent effect. Combinations with sub-MIC concentration of AmB and OTZ demonstrated indifference, with no evidence of antagonism at all tested concentrations of OTZ.

**Conclusion:**

We demonstrated *in vitro* partial synergy and indifference interactions of AmB and OTZ combination therapy against Tm using both the checkerboard and time-kill assays. Despite the lack of synergy, the absence of antagonism suggests that the AmB-OTZ combination may still improve efficacy, warranting further evaluation in *in vivo* and human studies.

**Disclosures:**

Thuy Le, MD, PhD, Gilead Science: Grant/Research Support

